# The atypical cyclin CNTD2 promotes colon cancer cell proliferation and migration

**DOI:** 10.1038/s41598-018-30307-x

**Published:** 2018-08-07

**Authors:** Abril Sánchez-Botet, Laura Gasa, Eva Quandt, Sara Hernández-Ortega, Javier Jiménez, Pau Mezquita, Miquel Àngel Carrasco-García, Stephen J. Kron, August Vidal, Alberto Villanueva, Mariana P. C. Ribeiro, Josep Clotet

**Affiliations:** 1 Faculty of Medicine and Health Sciences, Universitat Internacional de Catalunya, Barcelona, Spain; 2 Pathology Department, Hospital Universitari General de Catalunya, Sant Cugat del Vallès, Barcelona, Spain; 3 Department of Molecular Genetics and Cell Biology, The University of Chicago, Chicago, USA; 4 Department of Pathology, University Hospital of Bellvitge, Bellvitge Biomedical Research Institute (IDIBELL), CIBERONC, L’Hospitalet del Llobregat, Barcelona, Spain; 5 Xenopat S.L., Business Bioincubator, Bellvitge Health Science Campus, Barcelona, Spain; 6 Chemoresistance and Predictive Factors Group, Program Against Cancer Therapeutic Resistance (ProCURE), Catalan Institute of Oncology (ICO) Bellvitge Biomedical Research Institute (IDIBELL), L’Hospitalet del Llobregat, Barcelona, Spain

## Abstract

Colorectal cancer (CRC) is one of the most common cancers worldwide, with 8–10% of these tumours presenting a BRAF (V600E) mutation. Cyclins are known oncogenes deregulated in many cancers, but the role of the new subfamily of atypical cyclins remains elusive. Here we have performed a systematic analysis of the protein expression levels of eight atypical cyclins in human CRC tumours and several cell lines, and found that CNTD2 is significantly upregulated in CRC tissue compared to the adjacent normal one. CNTD2 overexpression in CRC cell lines increases their proliferation capacity and migration, as well as spheroid formation capacity and anchorage-independent growth. Moreover, CNTD2 increases tumour growth *in vivo* on xenograft models of CRC with wild-type *BRAF*. Accordingly, CNTD2 downregulation significantly diminished the proliferation of wild-type *BRAF* CRC cells, suggesting that CNTD2 may represent a new prognostic factor and a promising drug target in the management of CRC.

## Introduction

Colorectal cancer (CRC) is the third most commonly diagnosed malady and the fourth principal cause of cancer death in the world^1^. Different risk factors have been related to CRC progression, such as aging, chronic intestinal inflammation, or genetic alterations. Indeed, CRC is perceived as a set of diseases with distinct molecular signatures sharing the same clinical presentation, which can be classified according to their genetic profile. In this regard, the most frequently altered pathways in CRC include APC (in 80% of patients), the mutually exclusive RAS and BRAF (observed in 43% and 15% of the patients, respectively), as well as the Wnt pathway (in 93% of patients)^2^. Importantly, *BRAF* mutations are present in approximately 8–10% of the CRC patients^3,4^, who are not eligible for anti-EGFR therapy and are associated with poor clinical outcome^5,6^.

A fundamental feature of cancer is the deregulation of cell cycle control. The cyclin-dependent kinases (CDKs) are a group of serine/threonine kinases which control cell cycle progression through the interaction and activation of their regulatory partners, the cyclins^7^. Soon after their identification in 1982, cyclins have been associated with human cancers, with cyclin D1 garnering particular attention. Cyclin D1 is up-regulated in at least one-third of CRCs^8^, and contributes to CRC development and progression^9^. More recently, cyclin D1 overexpression was established as an unfavourable prognostic factor for CRC^10^. Likewise, overexpression of cyclin A is correlated with carcinogenesis and metastasis, and also constitutes a prognostic marker in patients with colorectal adenocarcinoma^11^.

While the majority of the studies conducted so far have addressed the function of canonical cyclins, the role of other proteins presenting the same characteristic “cyclin box”, a 150 amino-acids residue domain that defines the CDK binding^12–14^, remains largely unexplored. This group of cyclins appeared later as a result of the human genome sequence project, and were named “atypical” due to their structural specificities. Noteworthy, previous analysis of mRNA levels in CRC have not identified alterations in the expression of some of these atypical cyclins. Nevertheless, a correlation between gene amplification and the final protein levels of Cyclin A, B, D1 and D3 seems to be absent in CRC^8^, reflecting the importance of post-transcriptional regulation in the abundance of cyclin proteins family. Therefore, the investigation of the protein expression of atypical cyclins may allow the identification of new players in cell cycle regulation, which can be targeted to arrest tumour CRC cell proliferation.

In the present work, we monitored the protein expression of eight atypical cyclins in human CRC cell lines, as well as in resected CRC tumours, and identified CNTD2 as commonly upregulated in CRC. Studies in CRC cell lines and xenograft mouse models indicate that aberrant expression of CNTD2 may have functional significance, suggesting that CNTD2 represents an innovative drug target candidate in CRC.

## Results

### The protein level of CNTD2 and CCNO is increased in CRC tissues

**Figure 1:**
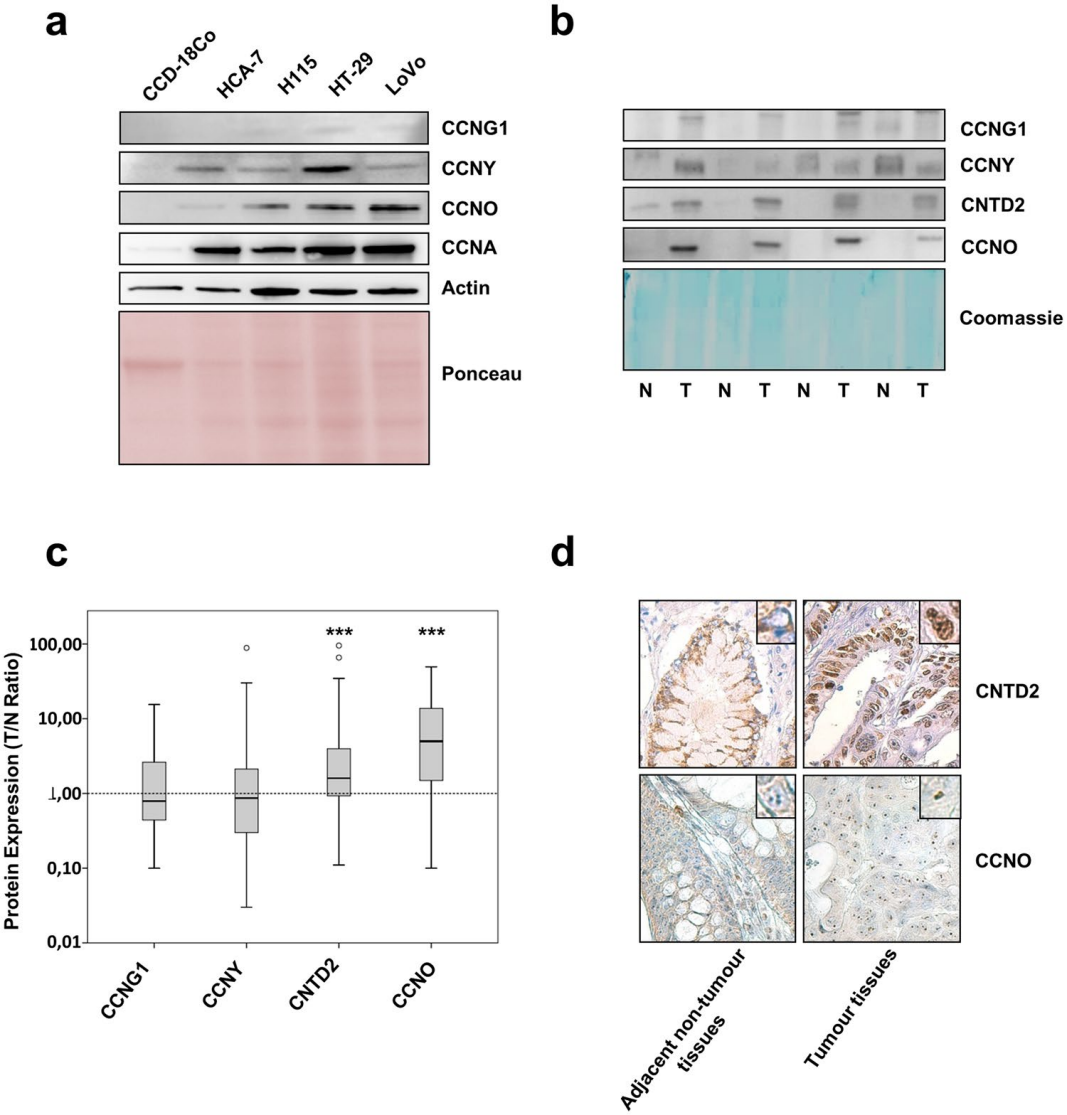
CCNO and CNTD2 are overexpressed in human colon cancer tissues. The protein screening of atypical cyclins was evaluated by western blot analysis. (**a**) Representative images of the expression of atypical cyclins in human colorectal cancer cell lines, HT-29, LoVo, HT115 and HCA-7, relative to colon normal fibroblasts, CCD-18Co. (**b**) Representative images of the atypical cyclins expression in normal (N) and tumour (T) FFPE human colon tissues. (**c**) Box plots of the expression ratio between tumour and normal colon tissues (T/N ratio) is presented. The whiskers indicate the range of the data and the horizontal bars represent the median (n = 55). ***P < 0.001 vs normal tissues, Wilcoxon test. (**d**) Representative immunohistochemistry images of human colon carcinoma samples stained with CNTD2 and CCNO antibodies. In both cases, positive protein expression was higher in tumour tissues. CNTD2 was localized to the nucleus whereas CCNO was localized to the nucleolar region of tumour cells (magnification 63x). Western blot images (**a** and **b**) have been cropped for clarity with full blots presented in Supplementary Fig. S5

To elucidate the potential role of atypical cyclins in CRC, we studied the expression of CCNG1, CCNG2, CCNI, CCNO, CCNY, CNTD1, CNTD2 and SPY1 in four colorectal cancer cell lines, LoVo, HT-29, HT115 and HCA-7, and compared it to the fibroblastic cell line from normal colon CCD-18Co. Taking into account that cyclins are mainly regulated by post-translational mechanisms and that the role played by these cyclins has not yet been revealed by the majority of high-throughput studies published so far, we decided to monitor the final protein levels as a measure of the expression of these genes. Therefore, only atypical cyclins with antibodies that have been previously validated were included in the present screening. The expression of the canonical cyclin A (CCNA) was used as a control and, as described, CCNA was up-regulated in cancer cell lines, relative to the normal colon cells (Fig. 1a), while the expression pattern of atypical cyclins was variable. The expression levels of CCNO were higher in tumour cell lines than in the normal one, while CCNY and CCNG1 exhibited higher expression in HT-29 cells (Fig. 1a). On the other hand, CNTD1, CNTD2, CCNG2, CCNI and SPY1 were not detected in any of the cell lines used. These results show that some of the atypical cyclins might be deregulated in CRC and that their expression in CRC is cell type-specific.

We then monitored the protein levels of atypical cyclins in 55 formalin-fixed and paraffin embedded (FFPE) samples from patients with colorectal adenocarcinoma, and their paired adjacent non-tumour colorectal tissue. The protein expression was monitored by western blot and the ratio between tumour and normal tissues (T/N ratio) was established after normalization with coomassie staining of the membrane. No differences were found regarding CCNG1 and CCNY expression in normal and tumour tissues, while the expression of CCNG2, CNTD1, SPY1 and CCNI was not detected. Remarkably, the average expression of CNTD2 and CCNO was significantly higher in cancer tissues compared to their normal counterparts (Fig. 1b,c) and, therefore, these cyclins were selected for further characterization in colon cancer cell lines.

In order to confirm the results obtained by Western blot, we examined six pairs of colon cancer and paired adjacent normal tissue by immunohistochemistry. We detected positive staining in tumour tissue for CNTD2, that was mainly localized in the nuclei (Fig. 1d), and for CCNO, which appeared concentrated in a location compatible with the nucleoli of tumour cells (Fig. 1d).

### CNTD2 overexpression enhances the proliferation of colon cancer cells

To evaluate the role of CCNO and CNTD2, these cyclins were overexpressed in two different cell lines: LoVo (a *BRAF* wild-type model of CRC cells) and HT-29 (a *BRAF* mutant cell line). Both cell lines were infected with an empty lentiviral vector (control) or with the lentiviral vector expressing CCNO or CNTD2. The expression of the constructs was evaluated by western blot using a Flag-tag fused to each cyclin, whereas GFP was used as reporter of the infection (Supplementary Fig. S1). The efficiency of infection was also monitored by FACS by the analysis of the GFP-expressing cells, revealing that 90% of LoVo and HT-29 cells were infected (data not shown).

**Figure 2:**
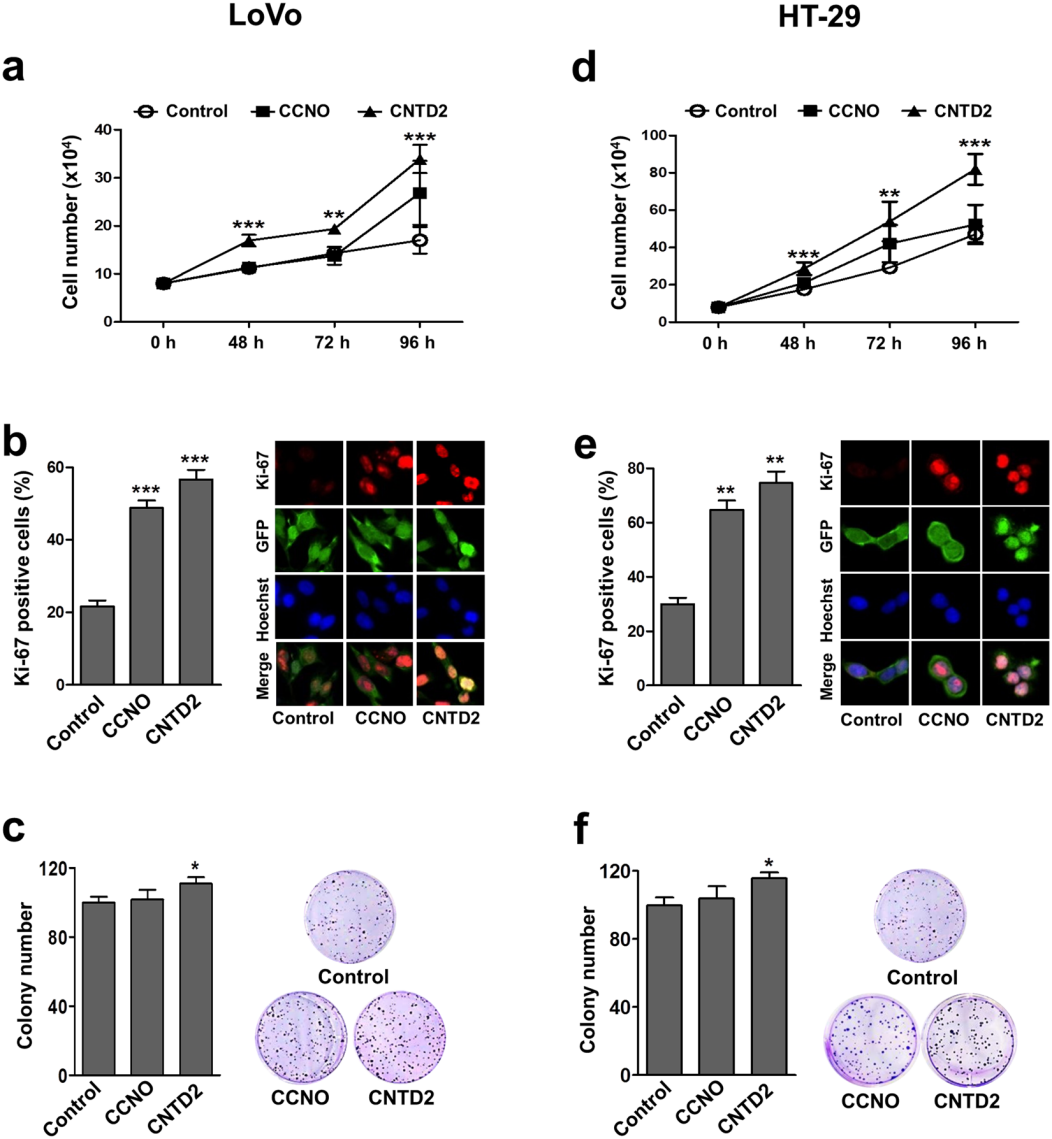
Overexpression of CCNO and CNTD2 increases the proliferation of CRC cells. LoVo and HT-29 cells were infected with empty lentiviral vector (control) or with the indicated cyclin-overexpressing construct. (**a**,**d**) CRC cell number was evaluated by counting. Columns represent the mean ± SEM of twelve independent experiments. **P < 0.01, ***P < 0.001 vs control, Mann-Whitney test. (**b**,**e**) The proliferation of CRC cells was evaluated by ki-67 immunostaining. Columns represent one representative experiment of four. In each experiment around 100 cells were analysed. **P < 0.01, ***P < 0.001 vs control, T-student test. (**c**,**f**) Efficiency of cell colony formation of CRC cells. Columns represent the mean ± SEM of eighteen independent experiments *P < 0.05 vs control, Mann-Whitney test. Results are expressed as a percentage of control.

While CCNO overexpression did not produce a clear effect on cell proliferation, the overexpression of CNTD2 increased the number of LoVo cells, the percentage of Ki-67-positive cells, as well as clonogenicity (Fig. 2a–c). The same results were recapitulated in HT-29 cells (Fig. 2d–f), supporting the idea that the role of CNTD2 on CRC cell proliferation is robust. Moreover, cell cycle analysis of asynchronous LoVo and HT-29 cells overexpressing CCNO and CNTD2 revealed that only the latter promoted significant cell cycle alterations on both cell types (Supplementary Fig. S1). Strikingly, there was no increase in S-phase in cells overexpressing CNTD2, which might be explained by the distribution of the proliferative effect among different phases of the cell cycle. Additionally, CNTD2 upregulation in HT-29 and LoVo cells did not significantly change the protein expression of caspase-9 (data not shown). These results point CNTD2 as a new important factor for CRC cell proliferation.

### CNTD2 overexpression promotes the migration of colon cancer cells

**Figure 3:**
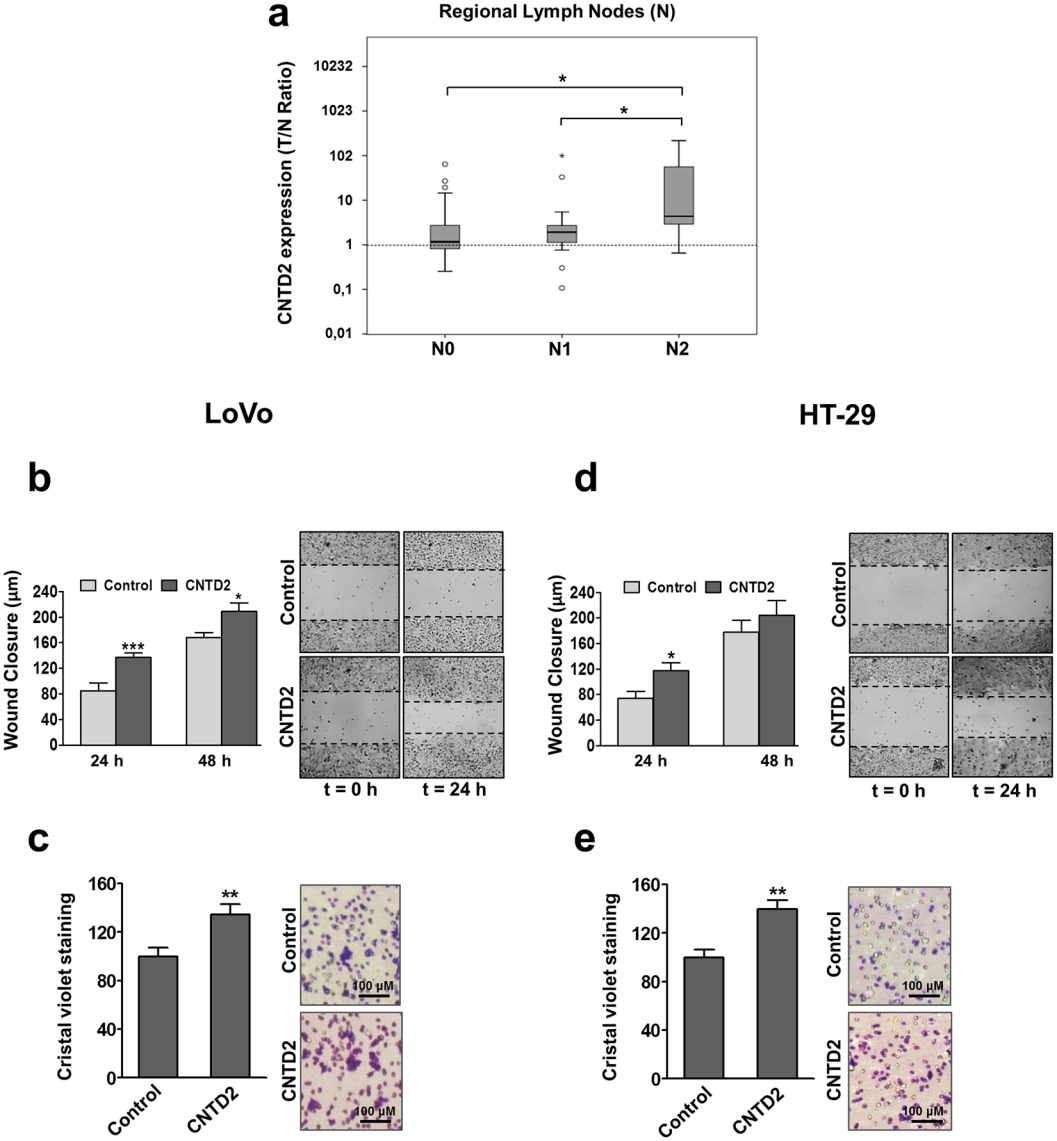
Overexpression of CNTD2 enhances the migration of CRC cells. (**a**) The T/N ratio of CNTD2 was evaluated in FFPE CRC tissue samples obtained from patients with different grade of regional lymph nodes (N), N0 (n = 25), N1 (n = 18) or N2 (n = 12) and is represented as box plots. *P < 0.05, Mann-Whitney test. (**b**,**d**) The migration of CRC cells was evaluated by the wound assay and the results are expressed as wound closure in µm. The columns represent the mean ± SEM of four independent experiments performed in triplicates. *P < 0.05 vs control, Mann-Whitney test. (**c**,**e**) CRC migration was monitored by the transwell assay and quantified by measuring the absorbance at 595 nm after acetic extraction of the crystal violet. The columns represent the mean ± SEM of four independent experiments performed in triplicate. **P < 0.01 vs control, Mann-Whitney test. Results are expressed as a percentage of control.

To investigate whether these atypical cyclins enhance the ability of CRC cells to establish distant metastasis, we determined the T/N expression ratio for all the cyclins under investigation in patients with different grades of regional lymph nodes (N0, N1 and N2). Of all the cyclins tested, we only found a positive correlation between CNTD2 expression and the different grades of regional lymph nodes (Fig. 3a). This result suggests that, apart from its role in CRC cell proliferation, CNTD2 upregulation might contribute to tumour migration and metastasis development. To confirm this hypothesis, we first monitored the effect of CNTD2 overexpression on CRC cell motility by the wound healing assay and observed that the overexpression of CNTD2 increased the rate of migration of LoVo cells in comparison with control (Fig. 3b). Accordingly, transwell assays confirmed greater migration of LoVo cells overexpressing CNTD2 to the lower chamber compared with control cells (Fig. 3c). Identical results were obtained when overexpressing CNTD2 in a second CRC cell line (Fig. 3d,e), indicating that CNTD2 may play an important role in promoting metastasis in CRC cells.

### CNTD2 upregulation promotes spheroid formation capacity and tumourigenicity

**Figure 4:**
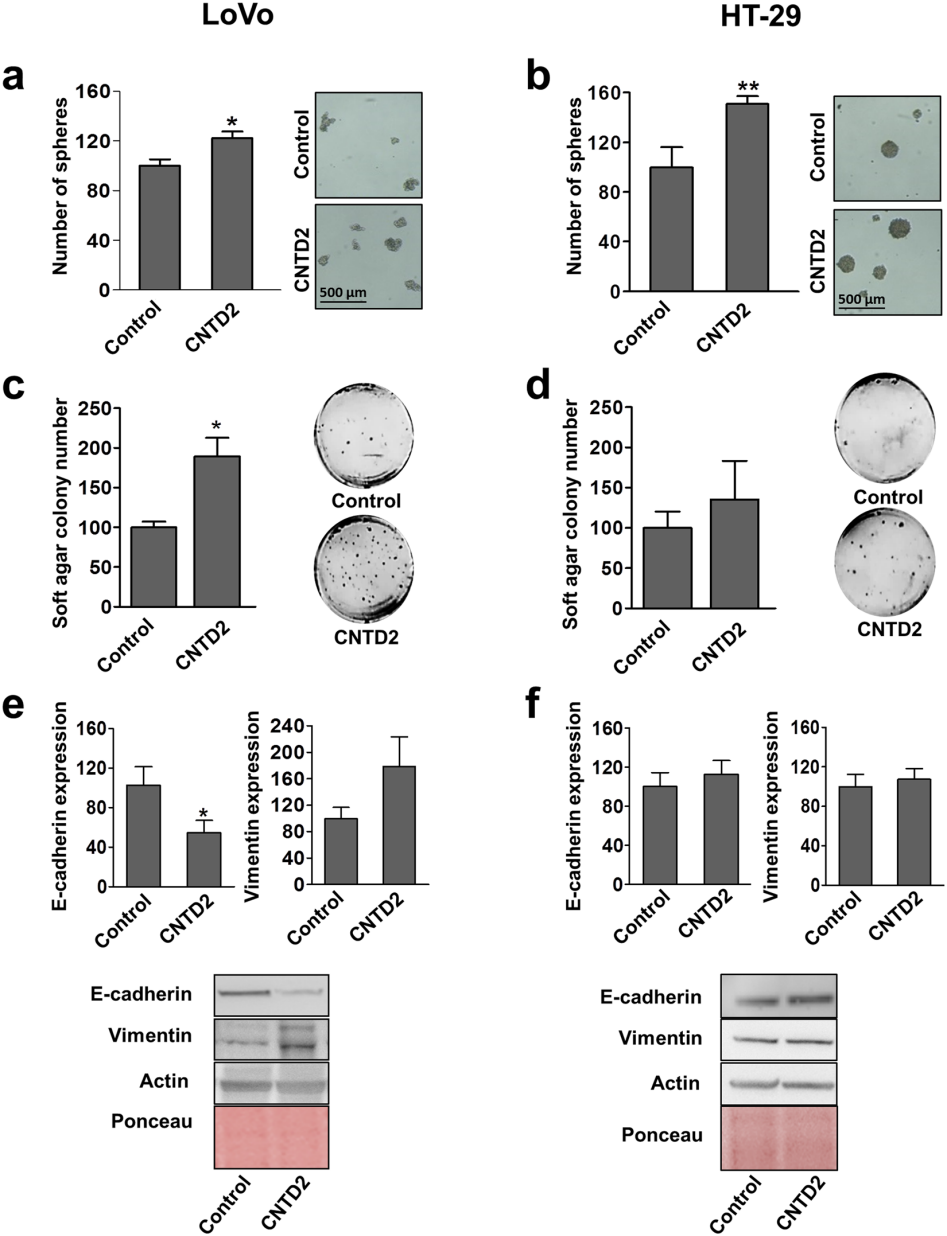
Overexpression of CNTD2 promotes spheroid formation capacity and tumourigenicity in CRC cells. (**a**,**b**) Efficiency of sphere formation of CRC cells in tumour sphere medium and ultra low attachment plates. Columns represent the mean ± SEM of six independent experiments performed in triplicates. *P < 0.05, **P < 0.01 vs control, Mann-Whitney test. (**c**,**d**) Effect of CNTD2 on anchorage-independent growth was determined by soft agar assay. Columns represent the mean ± SEM of five independent experiments performed in triplicates. *P < 0.05 vs control, Mann-Whitney test. (**e**,**f**) The effect of CNTD2 overexpression on EMT markers E-cadherin and vimentin was quantitated after normalization with Ponceau. Columns represent the mean ± SEM of eight independent experiments. *P < 0.05 vs control, Mann-Whitney test. Results are expressed as a percentage of control. Western blot images (**e** and **f**) have been cropped for clarity with full blots presented in Supplementary Fig. S6.

In order to travel throughout the body, colonize other tissues and establish distant metastases, tumour cells need to become anchorage-independent and acquire an epithelial-mesenchymal transition (EMT)-like phenotype^15^. To evaluate the involvement of CNTD2 in these processes, cells were cultured in suspension over low-attachment plates in serum-free medium containing growth factors to generate floating spheroid bodies. Our results show that CNTD2 overexpression enhances sphere formation capacity of both LoVo and HT-29 cells as compared to control (Fig. 4a,b), suggesting that CNTD2 may be involved in the self-renewal ability of CRC cells. On the other hand, anchorage-independent growth assays revealed that CNTD2 upregulation stimulated colony formation of CRC cells, although statistical significance was only found for LoVo cells (Fig. 4c,d). Next, we monitored the effect of CNTD2 upregulation on EMT markers by western blot. While CNTD2 overexpression in LoVo cells significantly decreased the levels of E-cadherin and showed a tendency to increase vimentin levels (Fig. 4e), we found no significant alterations regarding the expression of EMT in HT-29 cells (Fig. 4f).

### CNTD2 overexpression accelerates tumour growth *in vivo*

**Figure 5:**
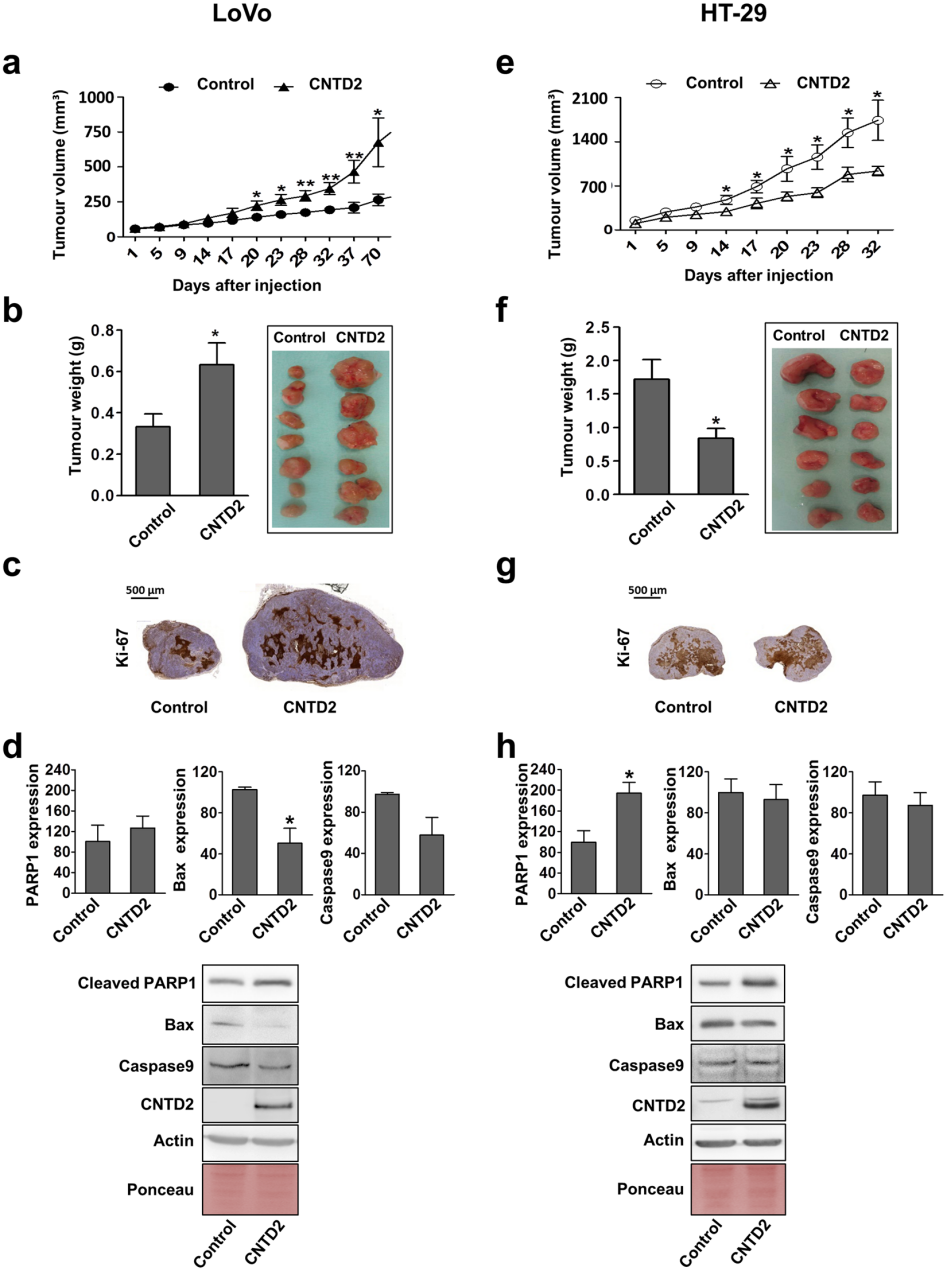
CNTD2 overexpression significantly enhances tumour growth *in vivo*. (**a**,**e**) The tumour volumes were measured at the indicated number of days after mice were transplanted with CRC cells infected with empty vector (control; n = 7) and CNTD2-overexpressing constructs (n = 7). *P < 0.05, **P < 0.01 vs control, Mann-Whitney test. (**b**,**f**) At day 70 mice were sacrificed and the tumours were resected and weighed. *P < 0.05, vs control, Mann-Whitney test. Representative images of resected tumours obtained from empty vector (control) and CNTD2 overexpressing cell-transplanted mice are shown. Results are expressed as a percentage of control. (**c**,**g**) Representative images of immunohistochemistry analysis of Ki67 in xenografts. Scale bar: 500 μm. (**d**,**h**) The effect of CNTD2 overexpression on markers of apoptosis was monitored by western blot in resected tumours of Lovo (n = 5) and HT-29 (n = 7) cells and quantitated after normalization with Ponceau. Columns represent the mean ± SEM.*P < 0.05 vs control, Mann-Whitney test. Results are expressed as a percentage of control. Western blot images (**d** and **h**) have been cropped for clarity with full blots presented in Supplementary Fig. S7.

Based on our cell-based results, we speculated that CNTD2 overexpression might promote tumour growth *in vivo*. Therefore, LoVo and HT-29 cells infected with empty vector (control) and with CNTD2-expressing constructs were subcutaneously injected into both flanks of nude mice. Tumours derived from LoVo cells overexpressing CNTD2 grew faster than those derived from cells infected with empty vector over time (Fig. 5a) and, at day 70 after injection, presented increased tumour size (Fig. 5b) and ki-67 staining intensity (Fig. 5c). Moreover, we investigated the effect of CNTD2 overexpression on three markers of apoptosis (cleaved PARP1, Bax and caspase 9) in resected tumours. In tumours derived from Lovo cells, we observed a trend to decrease caspase 9 expression and a significant decrease in Bax expression, suggesting that the increase in tumour size promoted by CNTD2 may reflect not only an increase in cell proliferation, but also a decrease in apoptosis (Fig. 5d). Strikingly, the opposite effect was observed in tumours derived from HT-29 cells, where CNTD2 overexpression was associated to decreased tumour growth (Fig. 5e) and volume (Fig. 5f), whereas ki-67 intensity (Fig. 5g) was similar to control in spite of the decreased tumour size. Noteworthy, in HT-29 cell-derived tumours, although no effect on Bax and caspase 9 expression was detected, we observed an increase in cleaved PARP1 (Fig. 5h).

**Figure 6:**
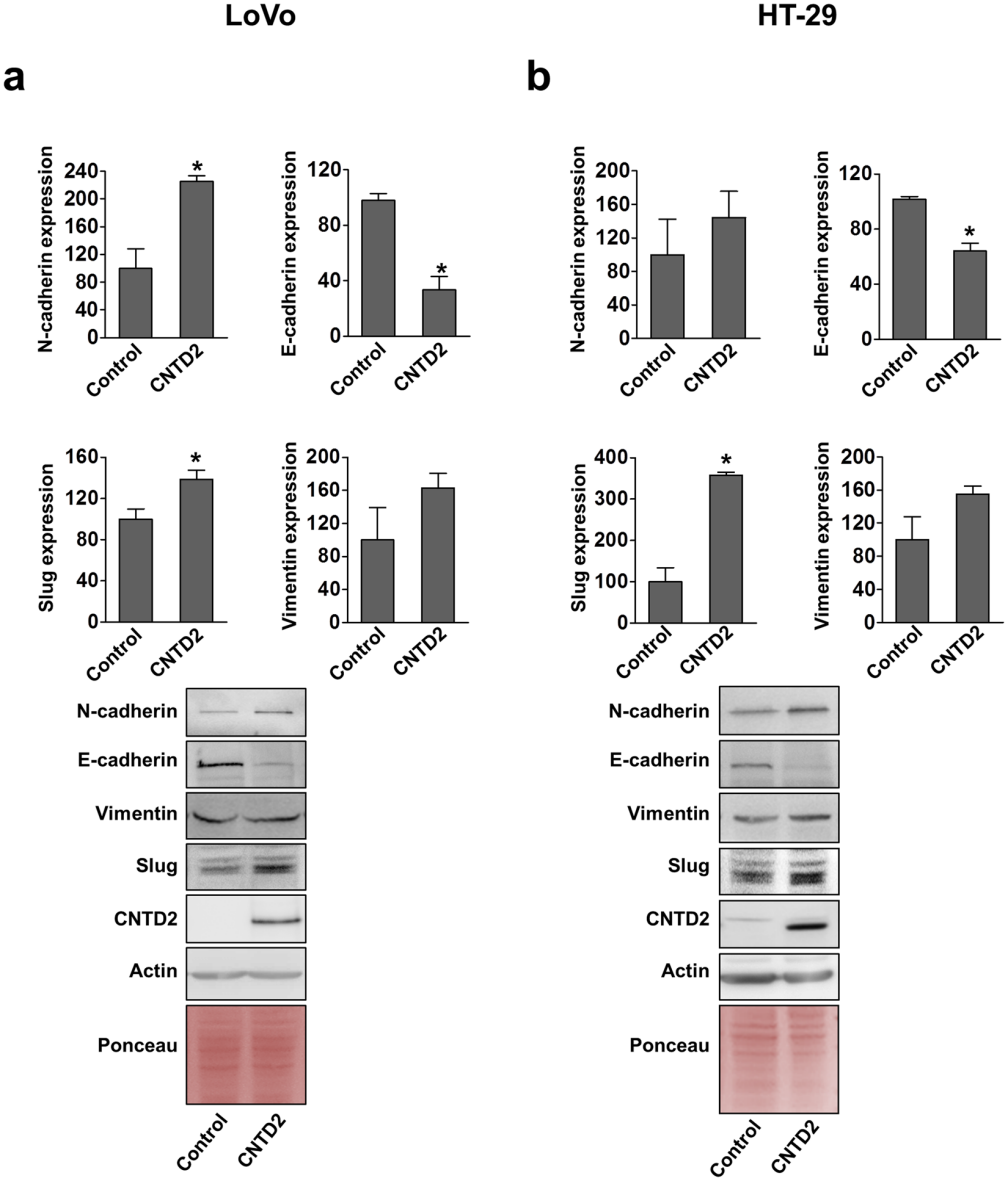
CNTD2 promotes EMT *in vivo*. The effect of CNTD2 overexpression on EMT markers was monitored by western blot in resected tumours and quantitated after normalization with Ponceau. Results are expressed as a percentage of control. (**a**) Columns represent the mean ± SEM of 5 independent experiments performed in Lovo xenotransplants. *P < 0.05 vs control, Mann-Whitney test. (**b**) Columns represent the mean ± SEM of 7 independent experiments performed in HT-29 xenotransplants. *P < 0.05 vs control, Mann-Whitney test. Western blot images have been cropped for clarity with full blots presented in Supplementary Fig. S8.

Afterwards, we investigated the expression of EMT markers, which relate with invasive characteristics, in resected tumours. In tumours derived from LoVo cells, CNTD2 overexpression significantly decreases E-cadherin expression and increases N-cadherin and Slug expression (Fig. 6a), whereas in tumours derived from HT-29 cells E-cadherin expression decreases and Slug expression increases (Fig. 6b), supporting a role for CNTD2 in EMT induction.

### CNTD2 as a prognostic factor and drug target

To investigate the prognostic significance of CNTD2 in CRC, Kaplan-Meier plots representing the overall survival of patients, were built using the PROGgeneV2 software. The level of CNTD2 expression did not affect the survival of CRC patients of all histological types (Supplementary Fig. S2A, p = 0.31). Similarly, CNTD2 mRNA levels did not significantly distinguish patients stratified by BRAF status (Supplementary Fig. S2, p = 0.069 or 0.528 for wild type or mutated BRAF, respectively). Whether protein levels are predictive remains to be determined.

**Figure 7:**
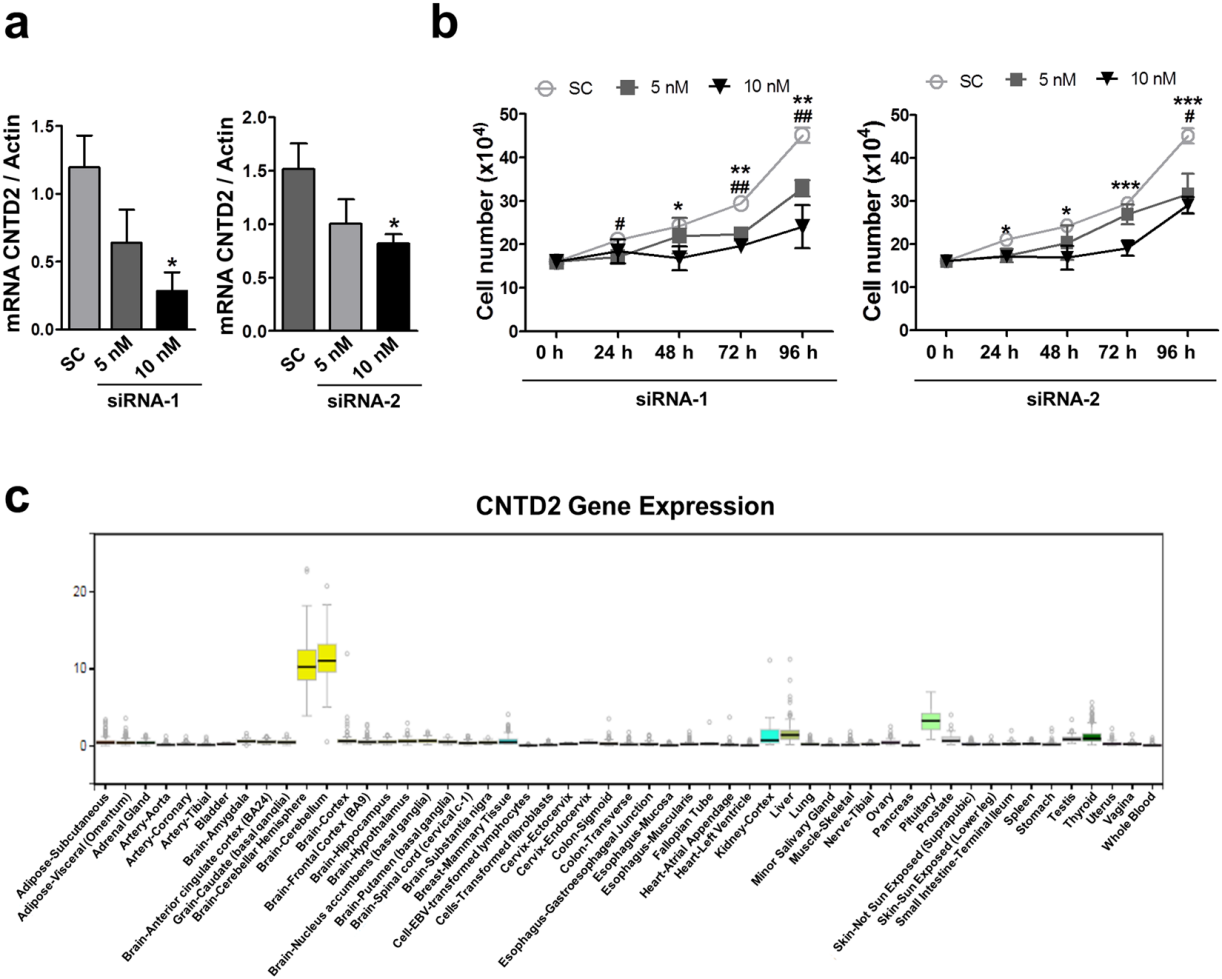
CNTD2 as a drug target candidate in CRC. LoVo cells were transfected with scrambled (Sc) RNA (10 nM) or with two different CNTD2-targeting siRNA (1 and 2) at 5 and 10 nM. (**a**) Down-regulation of CNTD2 mRNA was monitored by RT-qPCR. Columns represent the mean ± SEM of at least 4 experiments. *P < 0.05 vs scrambled, Mann-Whitney test. (**b**) LoVo cell number was determined by counting at the indicated time points after transfection. Values represent the mean ± SEM of at least four independent experiments performed in triplicates. *P < 0.05, **P < 0.01, ***P < 0.001 vs 10 nM, ^#^P < 0.05, ^##^P < 0.01 vs 5 nM, Mann-Whitney test. Results are expressed as a percentage of control. (**c**) CNTD2 gene expression in different tissues according to the GTEXPortal (https://www.gtexportal.org/home/).

As CNTD2 promotes CRC cell proliferation and migration, it is tempting to speculate that it represents an innovative therapeutic target. To investigate this hypothesis, CNTD2 expression was downregulated in LoVo cells using siRNA. The strategy used for siRNA validation is shown in Supplementary Fig. S3. Two different siRNA targeting CNTD2 (sequences 1 and 2) decreased mRNA levels (Fig. 7a) and cell number (Fig. 7b) in a dose-dependent manner as compared with cells transfected with scrambled control. Moreover, according to the GTEXPortal, CNTD2 is only expressed in the central nervous system (Fig. 7c), suggesting that CNTD2 can be a promising target candidate in CRC.

## Discussion

In spite of the improvements in diagnosis and therapy, many patients with CRC present recurrence and metastasis, which translates into poor prognosis and low survival rate^1,3,4^, highlighting the importance of screenings aimed at identifying new predictive and prognostic factors, as well as innovative pharmacological target candidates. In the present study, we performed a screening of the protein levels of eight atypical cyclins in CRC tissues, which revealed that some are deregulated and may be involved in CRC development and progression.

Our study detected that CCNO is upregulated at the protein level in human samples of CRC (Fig. 1b,c). However, the overexpression of CCNO did not increase the proliferation ability of LoVo and HT-29 cells, despite the increase in the percentage of ki-67-positive cells (Fig. 2b,e), revealing a cryptic role for this cyclin in CRC cell proliferation. Furthermore, according to the Kaplan-Meier Plotter database (http://kmplot.com/), CCNO upregulation is correlated with a reduced overall survival of lung and breast cancer patients (data not shown), which further supports that CCNO may be involved in the proliferation features of cancer cells. Further studies to elucidate the molecular role of this protein are now warranted.

In the present work, we show for the first time that CNTD2 is upregulated at the protein level in human samples of CRC (Fig. 1c,d), an important piece of evidence that remained undetected after both transcriptomic and proteomic analyses^16^. One may ask why the role of this gene has only now been uncovered. While the low level of cyclins expression may hamper proteomic approaches with high dynamic range mixtures, and the discrepancies between mRNA and protein levels challenge transcriptomic studies, CNTD2 was annotated as a gene quite recently^17^. Therefore, CNTD2 has not been included in the majority of microarray analyses and only with the development of RNA-seq technology, which allowed to identify unannotated transcripts, it started to be monitored in a few reports. Noteworthy, our observation that CNTD2 is upregulated at protein level in CRC is in line with the higher levels of mRNA reported by the Oncomine database (Supplementary Fig. S4).

The high expression level of CNTD2 in CRC tissues suggests that it might represent a new oncogenic driver, an observation that was supported by several facts, like its ability to increase CRC cell number (Fig. 2), the correlation between CNTD2 and tumour spreading (Fig. 3a), and by the fact that CNTD2 upregulation increased migration of CRC cells (Fig. 3b–e) and anchorage-independent growth (Fig. 4c,d). In agreement with our data, the analysis of the genomic landscape of patients with metastatic CRC (n = 349) conducted by the group of Meijer revealed that CNTD2 was among the genes more frequently amplified^18^, whereas the group of Kim reported that liver metastasis present higher expression of CNTD2 than the primary tumour from the same CRC patient^19^. Finally, the role of CNTD2 as a factor of malignancy in CRC is also in line with our previous results in lung cancer^20^ and, therefore, it is reasonable to speculate that CNTD2 might also be involved in metastasis development in other types of cancer as well. Indeed, according to the FIREBROWSE database, in addition to CRC, high levels of CNTD2 mRNA were found in breast, thyroid, and liver cancers, as compared to normal tissue (Supplementary Fig. S4). All these data converge to point CNTD2 as a new oncogenic driver in several types of cancer.

The aggressive phenotype induced by CNTD2 might reflect its ability to promote EMT in LoVo cells, as suggested by the decreased E-cadherin expression (Fig. 4e) and enhanced spheroid formation (Fig. 4a), which was further supported by the evaluation of EMT markers in resected tumours (Fig. 6a). Strikingly, CNTD2 upregulation did not affect the expression of EMT markers in HT-29 cells (Fig. 4f), although significant effects were detected *in vivo* (Fig. 6b), suggesting that EMT occurs later in time. These findings may reflect the fact that HT-29 cells were obtained from a primary tumour, whereas the LoVo cell line was derived from a fragment of a metastatic tumour nodule and, therefore, might possess some mesenchymal characteristics and start EMT more promptly. Moreover, the concept of epithelial plasticity^21^ supports the notion that the EMT is not working on a switch on and off basis, but that several intermediate EMT states occur in human cancers, and particularly in CRC, contributing to disease heterogeneity. Altogether, our results suggest that CNTD2 promotes a mesenchymal phenotype that may lead to tumour spreading, in agreement with the correlation with different grades of regional lymph nodes (Fig. 3a). At this point, more information on CNTD2 protein expression levels in patients with metastasis is needed to firmly establish a role for CNTD2 in metastasis development.

CNTD2 increased the growth of tumours derived from LoVo cells *in vivo* (Fig. 5a,b), in agreement with our observations using CRC cell lines, but surprisingly, the opposite effect was observed in tumours derived form HT-29 cells (Fig. 5e). Although other variables may contribute to these observations, both cell lines differ in their *BRAF* status (LoVo cells express wild-type *BRAF*, while HT-29 express mutated *BRAF*), suggesting that CNTD2 favours CRC cell proliferation in the short-term, but it may lead to different outcomes in the long-term based on BRAF status. In this regard, it is noteworthy that ki-67 intensity in tumours derived from HT-29 cells overexpressing CNTD2 is similar to control, whereas cleaved PARP1 increases (Fig. 5h). Therefore, the decreased growth rate associated to CNTD2 upregulation in this context may reflect an increase in apoptosis. CNTD2 mRNA levels did not affect prognosis, although analysis of protein levels will be required to make a definitive conclusion. We are currently working to understand the biological basis of the differential effect of CNTD2 overexpression on distinct genetic backgrounds, as well as the molecular mechanisms underlying the biological actions of CNTD2. Taking into account that CNTD2 belongs to the cyclins’ family it would be reasonable to expect that it interacts with a CDK and acts through the phosphorylation of some substrates. Studies are now underway to identify the interactors of this still orphan cyclin.

In conclusion, here we report that CNTD2 is upregulated in CRC, and promotes cell proliferation and migration, as well as increased tumour growth *in vivo*. The decrease in cell number observed when CNTD2 is downregulated (Fig. 7b), along with the fact that CNTD2 is poorly expressed in healthy tissues outside the central nervous system (Fig. 7c), supports the development of new therapeutic strategies targeting CNTD2 for the management of CRC.

## Materials and Methods

### Clinical samples

CRC tissues and paired-adjacent non-tumour tissues were obtained from 55 patients diagnosed at the Hospital General de Catalunya and Hospital Universitari Sagrat Cor. Samples were selected based on tumour content (minimally 80%). For inclusion in this study, a patient must have had a diagnosis of primary colon cancer and must had received no chemotherapy or radiation therapy before surgery. The samples were fixed in formaldehyde and embedded in paraffin (FFPE) according to routine procedures at these hospitals. The tumours were histologically classified, graded and staged according to the World Health Organization criteria. Patients’ characteristics included in this study are summarized in Supplementary Table S1. This study was approved by the Ethics Committee of idcsalud Hospital General de Catalunya (CEIC) and carried out in accordance with the approved guidelines. The requirement to obtain informed consent was waived by the Ethics Committee because of the retrospective nature of the study, but an informed consent was obtained from living patients.

### Cell lines and Reagents

Human colorectal cancer cell lines, LoVo, HT-29, HT115 and HCA-7, and normal colorectal cell line CCD-18Co, were cultured in Dulbecco’s Modified Eagle Medium (DMEM; Sigma-Aldrich) supplemented with 10% fetal bovine serum (FBS; Sigma-Aldrich), 1% glutamax (Biowest) and 1% penicillin/streptomycin (Sigma-Aldrich). Cells were grown in humidified air at 37 °C and 5% CO_2_. Cells were used for no more than 5 passages after thawing. Mycoplasma contamination was monitored periodically.

### Western blot analysis

Protein was extracted from FFPE tissues, human cell lines and resected tumours, and western blot analysis was performed as previously described^20^. The primary antibodies used are listed in Supplementary Table S2 and were previously validated^20^.

### Immunohistochemistry

Immunohistochemical staining of FFPE clinical samples was performed as described elsewhere^20^. Polyclonal antibodies against CNTD2 (ab179781, Abcam) and CCNO (ab47682, Abcam) were used at a dilution of 1:100. For the analysis of ki-67 in tumours from xenografts, FFPE tissue sections were stained using the Dako Omnis (Dako, Agilent Technologies, Inc.) according to the manufacturer’s instructions and antigen retrieval was performed using EnVision FLEX Target Retrieval Solution, High pH (Dako, Agilent Technologies, Inc.). Tissue sections were subsequently incubated with primary antibody (GA626, Ki-67, clone MIB-1, ready-to-use, Dako, Agilent Technologies, Inc.) and the chromogenic visualization was performed using EnVision FLEX/HRP (Dako, Agilent Technologies, Inc.). Appropriate controls were stained concurrently to validate the procedure.

### Viral cloning, transduction and infection

Lentiviral vectors were prepared as previously described^20^. The viral titer was determined by infecting HEK293-T cells with serial dilutions of virus and the optimal MOI was determined for the LoVo and the HT-29 cells, which was 30 MOI and 40 MOI, respectively. For overexpression studies, LoVo and HT-29 cells were cultured and, 24 h later, half of the medium was removed and the cells infected. Supplemental medium was added on the following day and after 4 days the cells were analysed.

### Cell counting

Cells were plated in 24-well plates at a density of 20,000 cells per well and 24 h later cells were infected. At the indicated time points, cells were washed with PBS and trypsinized for counting with the haemocytometer under a 10x objective.

### Ki-67 immunofluorescence

LoVo and HT-29 cells were seeded on poly-lysine coated coverslips in 24-well plates at a density of 15,000 cells, and the staining performed as described before^20^.

### Colony formation assay

Single LoVo and HT-29 cells were plated in 6-well plates at a density of 300 and 200 cells per plate, respectively. After 24 h, cells were infected, incubated for 15 days to allow colony formation, stained with 0.1% crystal violet and manually counted.

### Wound-healing and transwell migration assay

The wound healing assay was performed as described elsewhere^20^, except that cells were seeded at a density of 1 × 10^6^ of cells per well.

Migration assays were carried out as previously described^20^ with minor modifications. Cells were seeded at a density of 200,000 cells per well and allowed to migrate to the bottom chamber for 24 h.

### Sphere formation assay

Single cells were seeded on ultra-low attachment 24-well plates (Corning) at a density of 5,000 cells per well and incubated for 7 days in serum-free medium (DMEM/F12) supplemented with B27 (17504-044, Gibco, Thermo Fisher Scientific), EGF (20 ng/ml, AF-100-15 PeproTech) and FGF (20 ng/ml, 100-18B PeproTech). The number of spheres with a diameter >50 µm was quantified under the 4x objective of the microscope.

### Anchorage-independent growth assay

Six-well plates were pre-coated with complete medium containing 1% low melting agarose. Afterwards, 800 cells per well were mixed with culture medium containing 0.6% agar to a final concentration of 0.3%. The cell suspension was added to the precoated bottom agar gel, and after solidification, each well was covered with complete medium, which was refreshed every 3 days. After 21 days, images were taken using the GeneSnap (Syngene) software and spheres were counted using GeneTools (Syngene).

### siRNA Transfection and RT-qPCR Analysis

LoVo cells were seeded in 48-well plates at a density of 3 × 10^4^ cells/well. The following day, CNTD2 siRNA (SR312715A and SR312715B, OriGene) and the negative control (SR30004) were transfected using Lipofectamine 2000 (12566014, Thermo Fisher Scientific). At the indicated time points, cells were counted as described above and the RNA extracted for further analysis. The validation of the siRNA sequences was carried out as shown in Supplementary Fig. S3.

Experiments were conducted in parallel for total RNA extraction using the Nucleospin RNA isolation kit (740955, Macherey-Nagel). Complementary DNA (cDNA) was synthetized from total RNA using a reaction mixture composed of RT buffer (F88903, Lucigen), dNTP (D7295, Sigma), Random RT primer (309080, Exiqon), M-MuLV reverse transcriptase (30222-, bioNova), and Reverse transcriptase (30281-1, bioNova). The mixture was incubated at 37 °C for 1 h. Quantitative PCR was performed using SYBR Green I Master Mix (1725271, BioRad). The obtained values were normalized relative to the housekeeping gene 18S and calculated according to the 2^−ΔΔCt^ method. The following primers were used: CNTD2 FW (5′-CTGGTGGTAGACTGGCTGGT-3′), CNTD2 RV (5′-AGCACGCACTCTTCCATTTT-3′), Actin FW (5′-TCCACCTTCCAGCAGATGTG-3′), and Actin RV (5′-GCATTTGCGGTGGACGAT-3′).

### Tumour xenografts

3 × 10^6^ CRC cells were soaked in 100 μl of Matrigel (BD Biosciences) and subcutaneously injected into each flank of 5-weeks-old athymic nude male mice (Crl:NU-Foxn1nu) (n = 7 in each group). Tumour growth was monitored every 3 days, and the length (L), the width (W), and length (L) were measured until 9 weeks after injection. After euthanasia at day 70, the tumours were resected and weighed (g), and the tumour volume (mm^3^) was estimated from the formula V = π/(6 × L × W2). This study was approved by the IDIBELL Animal Care Committee and the methods were carried out in accordance with the approved guidelines.

### Statistical analysis

Data are presented as the mean ±  standard error of the mean (SEM) with P-values: ***P < 0.001; **P < 0.01; *P < 0.05 considered to indicate significant differences. Statistical significance was determined using the Mann-Whitney test for the non-parametric data or T-student for the parametric ones. To compare the expression ratio between tumour/normal tissues the Wilcoxon test was used. Statistical analyses were conducted using GraphPad Prism 5 and the Statistical Package for the Social Sciences (SPSS) 21.

## Electronic supplementary material

Supplementary Information
