# Supplementary material for: The atypical cyclin CNTD2 promotes colon cancer cell proliferation and migration
Source: Sci Rep. 2018 Aug 7;8:11797. doi: 10.1038/s41598-018-30307-x (PMC6081371; doi:10.1038/s41598-018-30307-x)
Supplement: Supplementary file 1 — Supplementary Information [file 41598_2018_30307_MOESM1_ESM.pdf]

The atypical cyclin CNTD2 promotes colon cancer cell proliferation and migration

Abril Sánchez-Botet[&], Laura Gasa[&], Eva Quandt, Sara Hernández-Ortega, Javier Jiménez, Pau Mezquita, Miquel Àngel Carrasco-García, Stephen J. Kron, August Vidal, Alberto Villanueva, Mariana P.C. Ribeiro[*], Josep Clotet[*]

[&]Contributed equally
*Corresponding authors

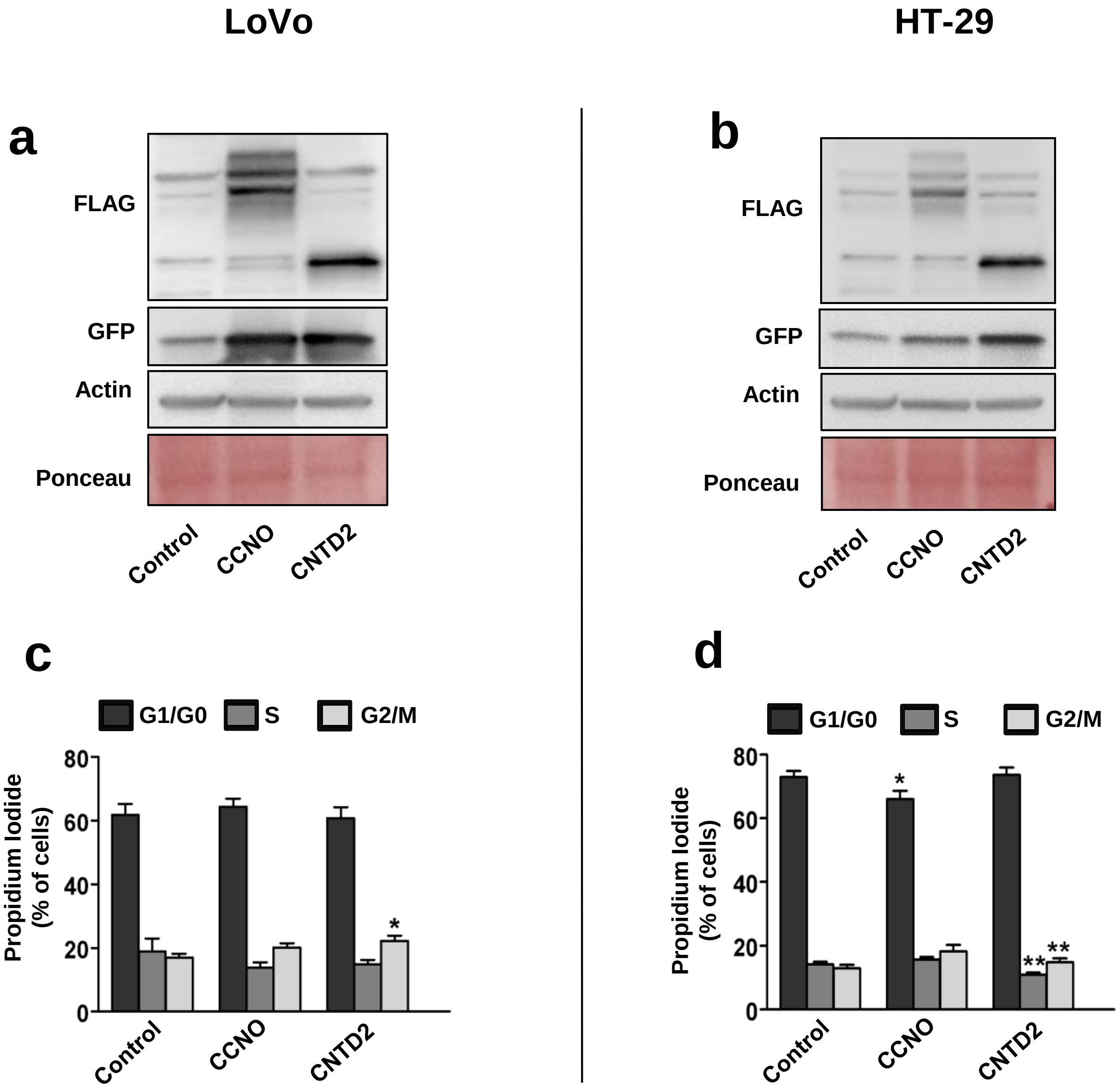

Supplementary Figure S1. Overexpression of CCNO and CNTD2 in CRC cells. LoVo and HT-29 cells were infected with empty lentiviral vector (control) or with the indicated cyclin-overexpressing construct. (a, b) Western blot analysis confirmed cell infection. Flag-tagged cyclins were detected with an anti-flag antibody at the expected band size and GFP was used as a reporter for lentivirus infection. (c, d) The cell cycle pattern was analysed by Propidium Iodide staining and FACS analysis. Columns represent the mean ± SEM of six independent experiments. *P < 0.05, **P < 0.01 vs control, Mann-Whitney test. Results are expressed as a percentage of control. Western blot images (a and b) have been cropped for clarity with full blots presented in Supplementary Fig. S9.

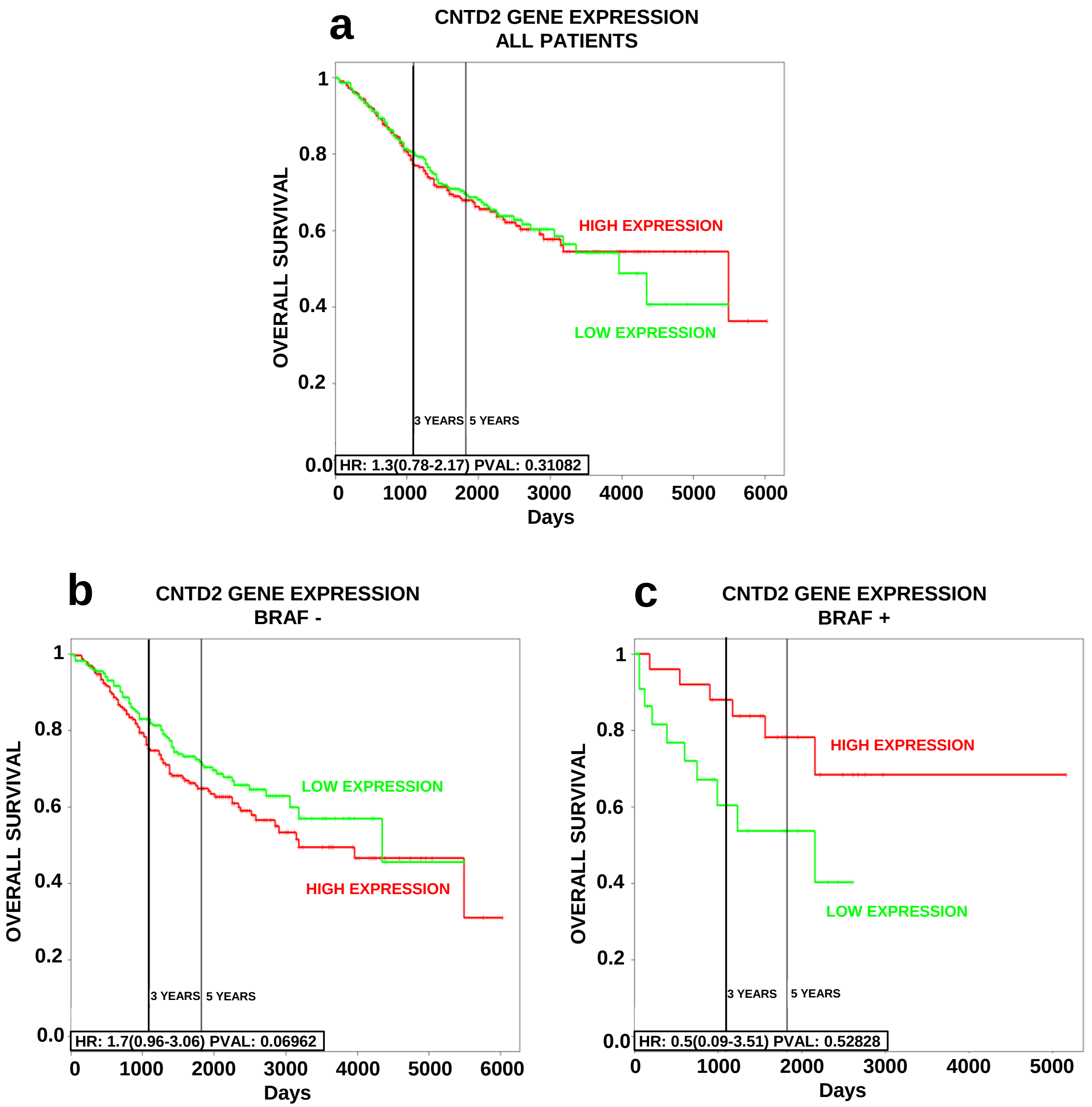

Supplementary Figure S2. Prognostic value of CNTD2 on CRC cancer survival. Kaplan-Meier plots were built using the PROGgeneV2 software (http://watson.compbio.iupui.edu/chirayu/proggene/database/?url=proggene) and choosing the GSE39582 database corresponding to Marisa (n=578). Graphs represent the overall survival of CRC patients (a) of all histological types, (b) with negative BRAF mutation (n=282) and (c) positive BRAF mutation (n=29) expressing different levels of CNTD2. Red coloured lines represent the patients with high gene expression, while green coloured lines represent patients with low gene expression. The P-values are shown on each graph.

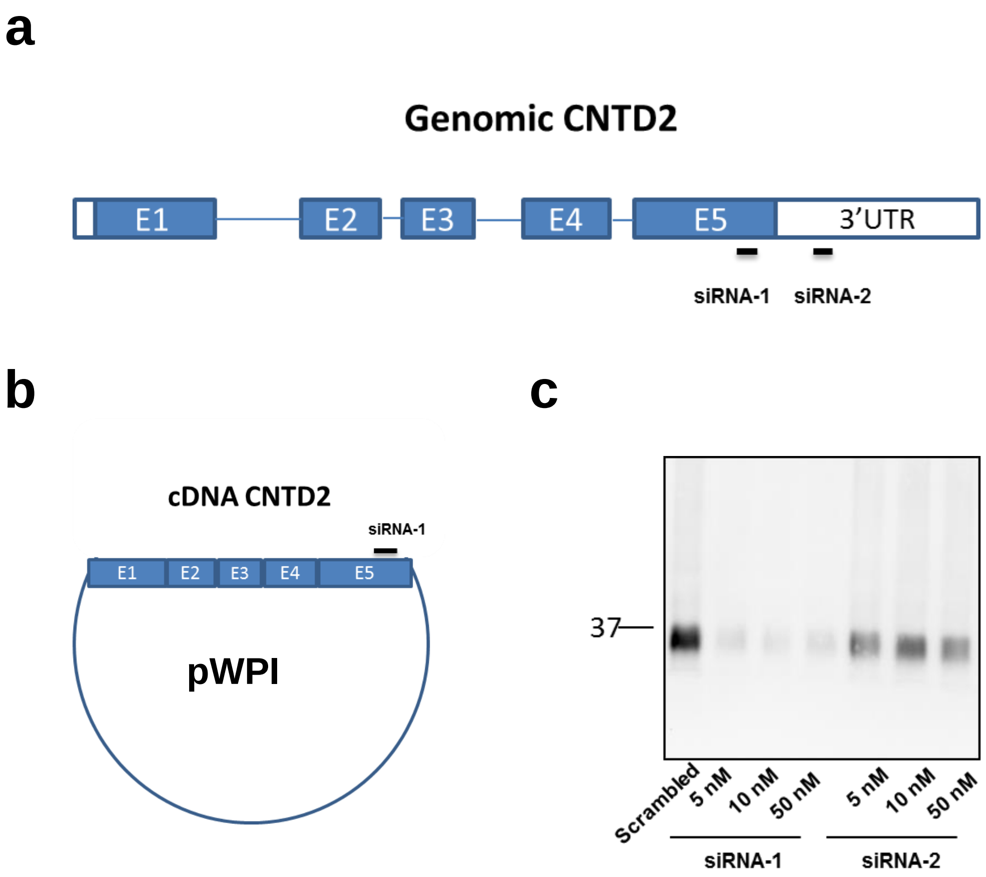

Supplementary Figure S3. CNTD2 siRNA validation. (a) Representation of genomic CNTD2 and the relative position of the siRNA sequences used: one targeting exon 5 (siRNA-1) and the other targeting the 3'UTR (siRNA-2). (b) Diagram of the CNTD2-expression construct used for cell infection, where only the target sequence of siRNA-1 can be found. (c) WB analysis demonstrates the specificity of siRNA-2 in A549 cells infected with the CNTD2-expression construct shown in (B).

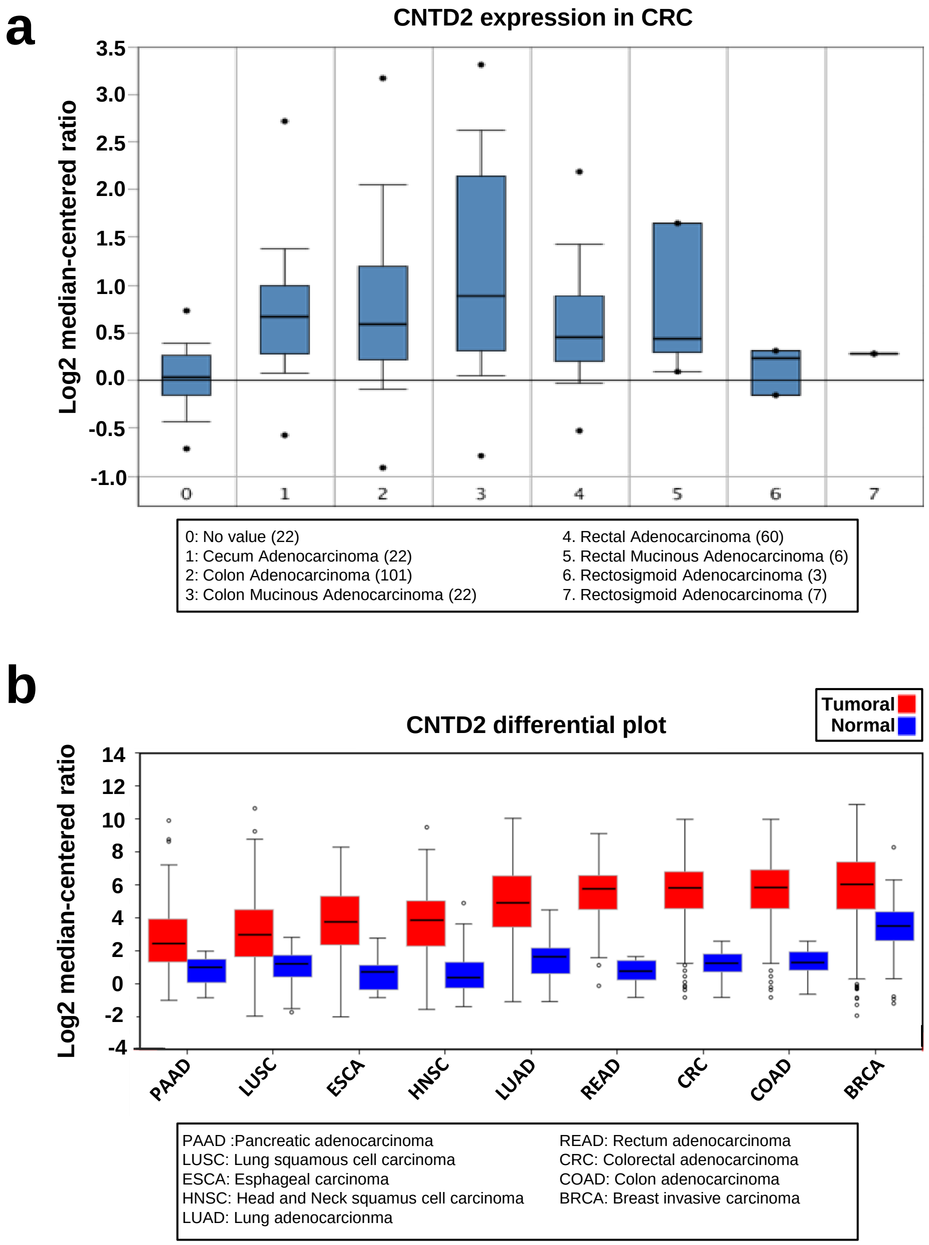

Supplementary Figure S4. Differential expression of CNTD2 in human normal and cancer tissues. (a) CNTD2 mRNA levels in CRC cancer samples of different histological types according to the Oncomine database (http://oncomine.org/) (b) Human cancers with more pronounced differences between tumour tissues and normal counterparts regarding the levels of CNTD2 mRNA according to the FIREBROWSE database (http://firebrowse.org/).

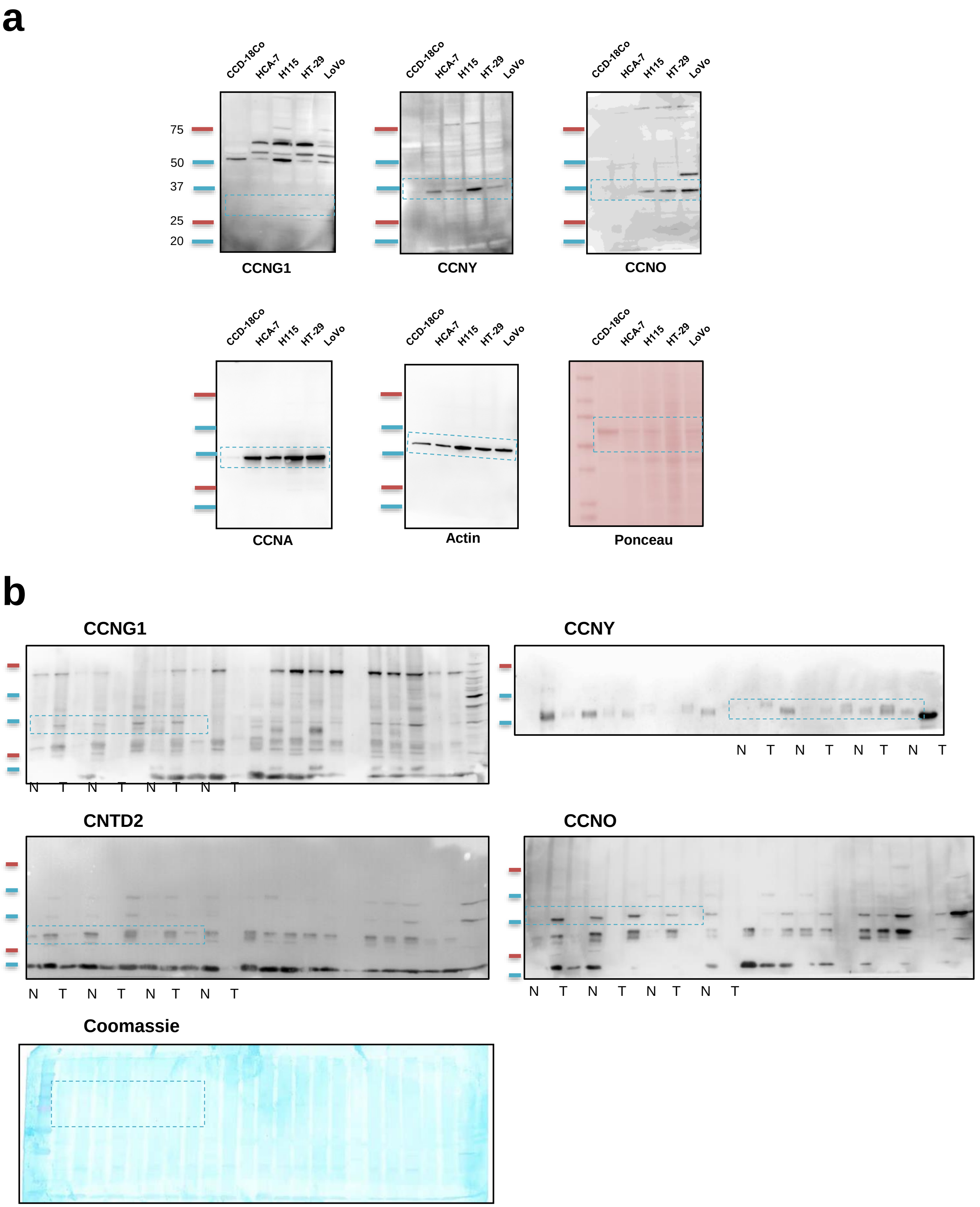

Supplementary Figure S5. Full blots from Figure 1a and 1b.

(a) Representative images of the expression of atypical cyclins in human colorectal cancer cell lines, HT-29, LoVo, HT115 and HCA7, relative to colon normal fibroblasts, CCD-18Co. (b) Representative images of the atypical cyclins expression in normal (N) and tumour (T) FFPE human colon tissues. Dashed rectangles indicate the areas presented in the main figures.

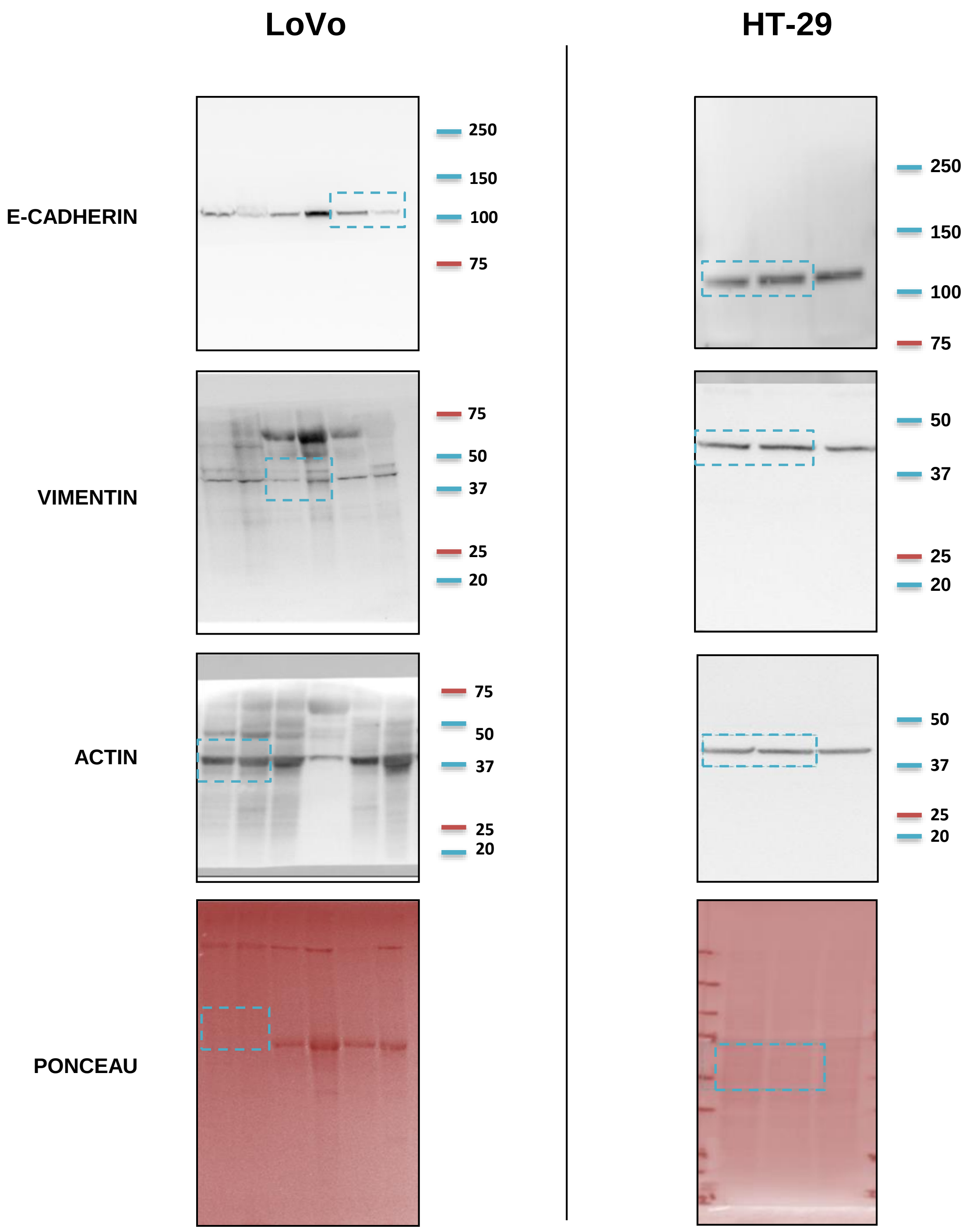

Supplementary Figure S6. Full blots from Figure 4e and 4f.

(e, f) The effect of CNTD2 overexpression on EMT markers E-cadherin and vimentin was quantitated after normalization with Ponceau. Dashed rectangles indicate the areas presented in the main figures.

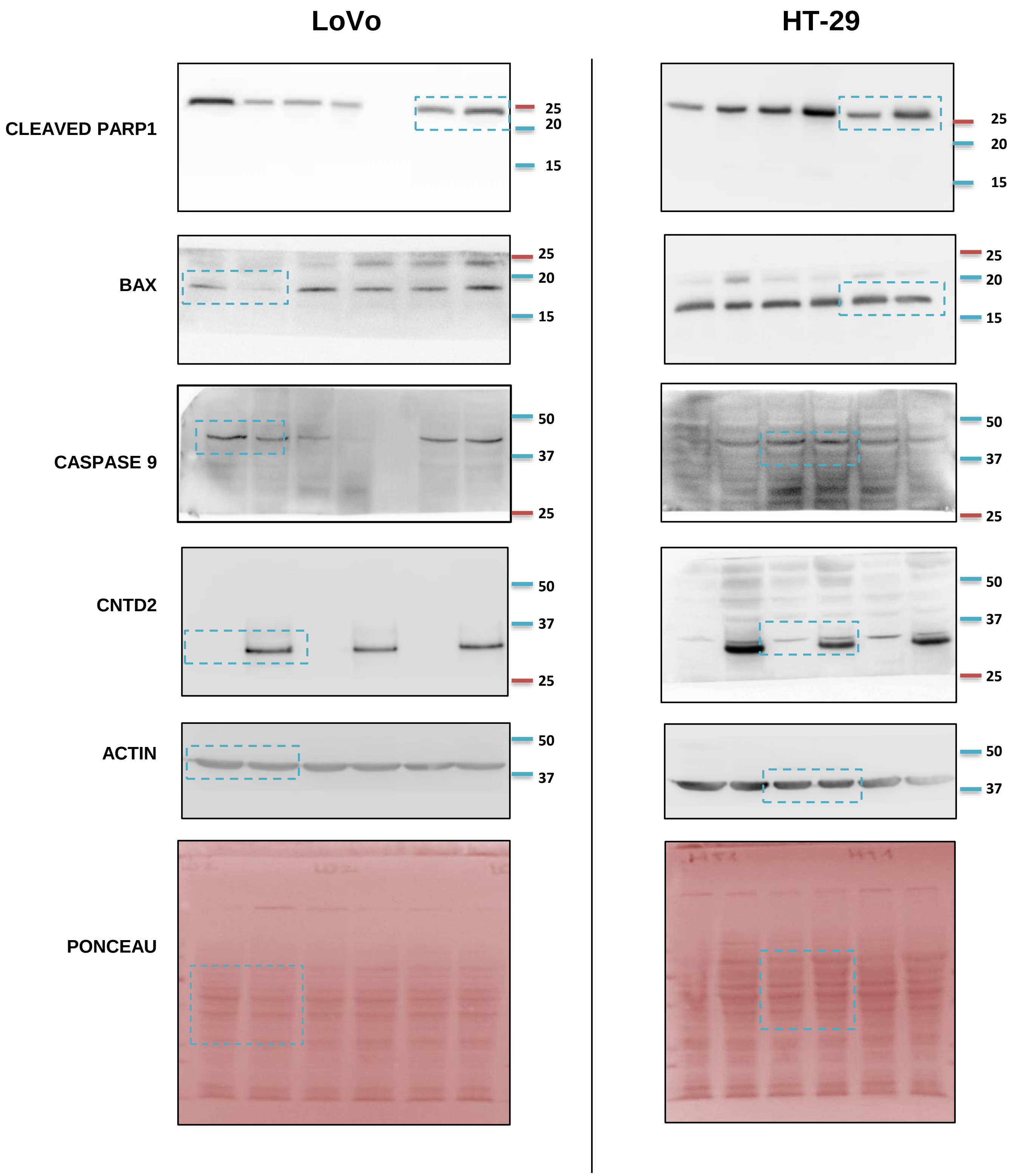

Supplementary Figure S7. Full blots from Figure 5d and 5h.

(d, h) The effect of CNTD2 overexpression on markers of apoptosis was monitored by western blot in resected tumours and quantitated after normalization with Ponceau. Dashed rectangles indicate the areas presented in the main figures.

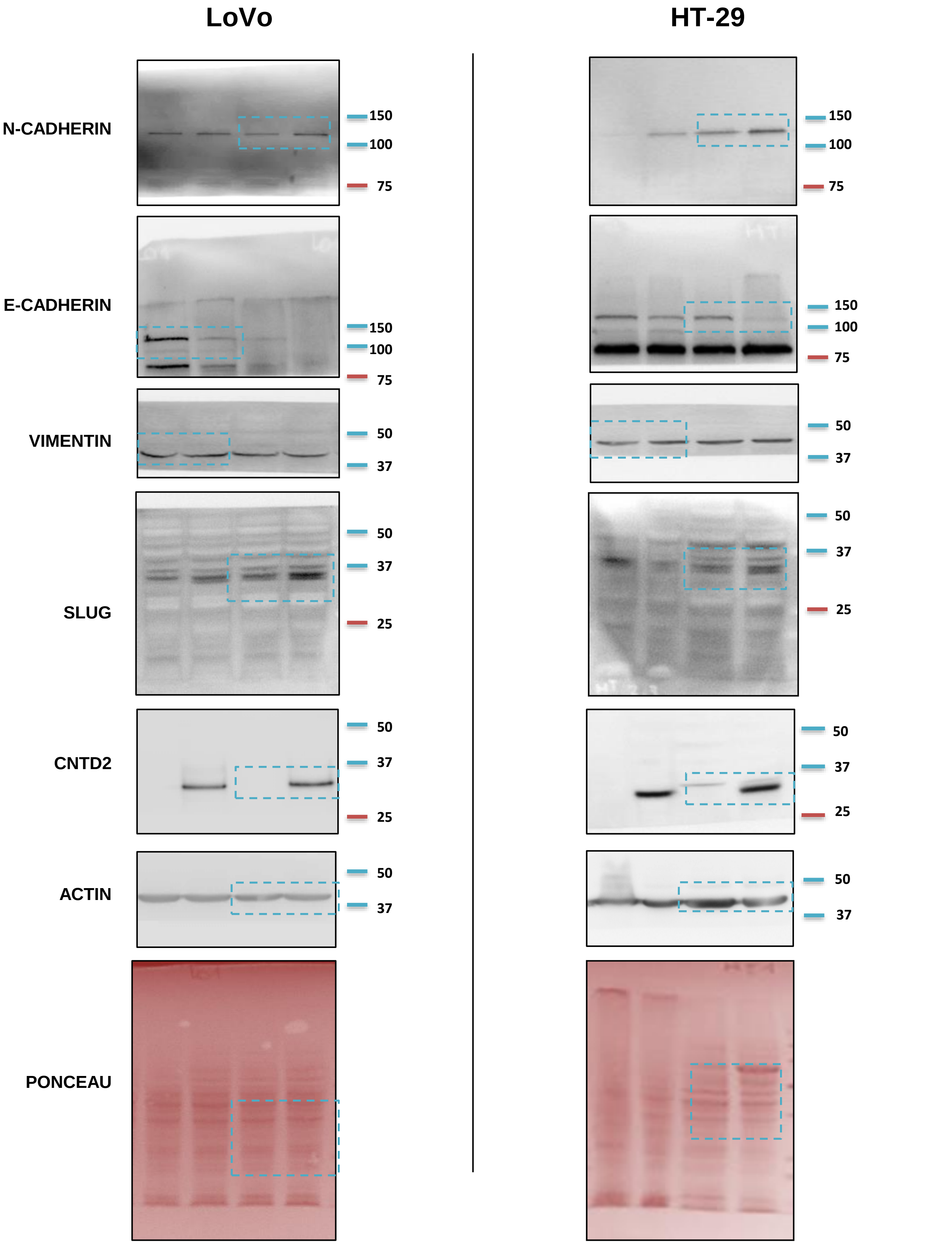

Supplementary Figure S8. Full blots from Figure 6.
The effect of CNTD2 overexpression on EMT markers was monitored by western blot in resected tumours and quantitated after normalization with Ponceau. Dashed rectangles indicate the areas presented in the main figures.

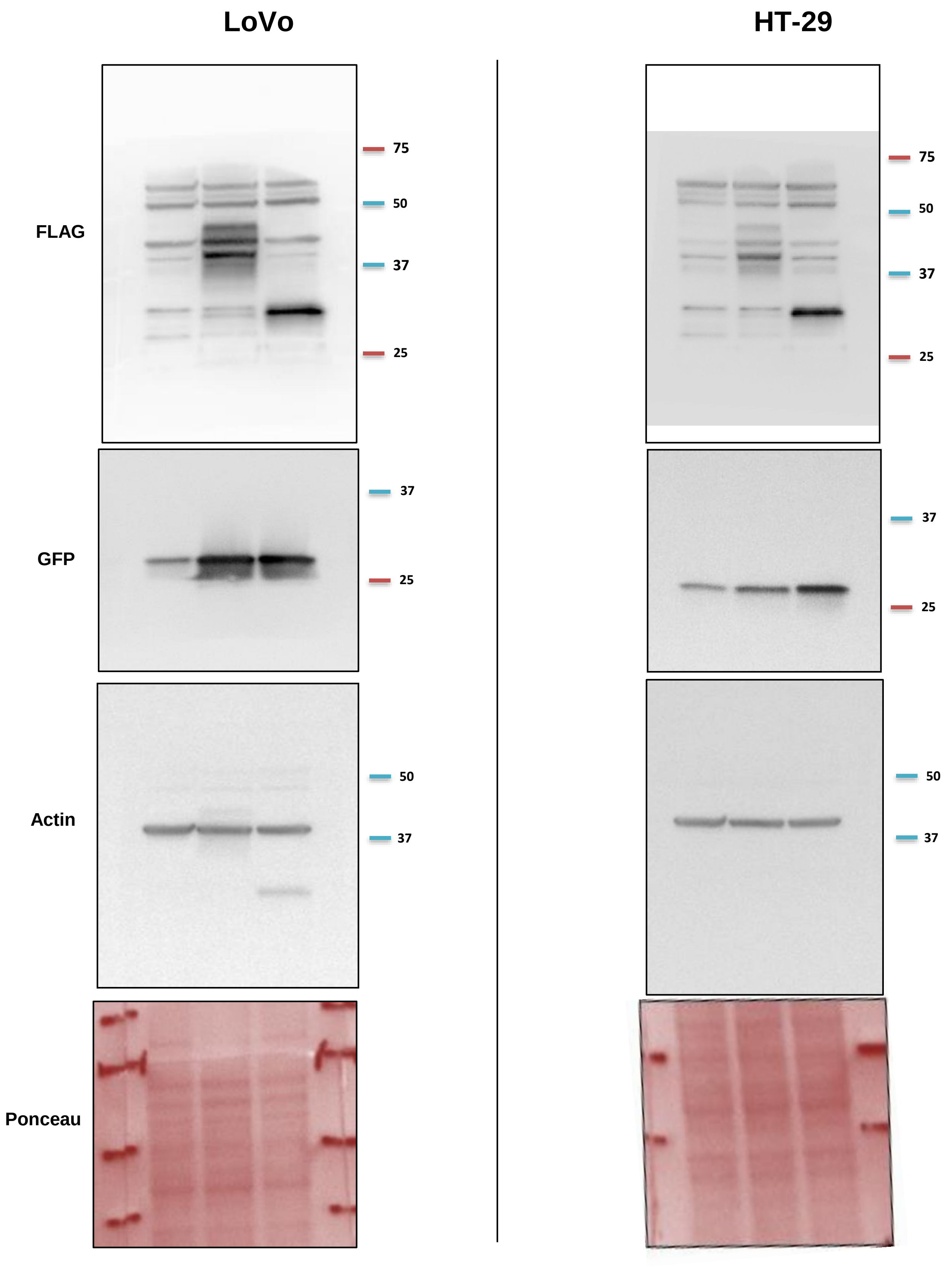

Supplementary Figure S9. Full blots from Supplementary Figure S1a and S1b.

(a, b) Western blot analysis confirmed cell infection. Flag-tagged cyclins were detected with an anti-flag antibody at the expected band size and GFP was used as a reporter for lentivirus infection.

		Number (%)
Histologic Grade		
	Low	49 (89.1%)
	High	6 (10.9%)
Ganglia +		30 (54.5%)
Stage		
	I	-
	II	22 (40%)
	III	26 (47.3%)
	IV	7 (12.7%)
Sex		
	Female	30 (54.5%)
	Male	25 (45.5%)
Age (years)		71.1 (34 - 92)
Side tumour		
	Left	19 (34.5%)
	Right	31 (56.4%)
	Transverse	5 (9.1%)

Supplementary Table S1. Clinicopathologic features of the subjects included in the study (n=55).

Cat. No	Antibody	Species	Dilution	Company
2956	GFP	Rabbit	1:500	Cell Signaling
ab179781	CNTD2	Rabbit	1:2,000	Abcam
ab47682	CCNO	Rabbit	1:500	Abcam
ab114086	CCNY	Rabbit	1:1,000	Abcam
ab126998	CNTD1	Rabbit	1:6,000	Abcam
sc-320	CCNG1	Rabbit	1:200	Santa Cruz Biotechnology
sc-7266	CCNG2	Goat	1:500	Santa Cruz Biotechnology
sc-5547	CCNI	Rabbit	1:200	Santa Cruz Biotechnology
sc-751	CCNA	Rabbit	1:1000	Santa Cruz Biotechnology
NB100-25-21	SPY1	Rabbit	1:500	Novusbio
ab32503	Bax	Rabbit	1:1000	Abcam
sc-73548	Caspase-9	Mouse	1:200	Santa Cruz Biotechnology
ab32064	Cleaved PARP1	Rabbit	1:1000	Abcam
sc-1500	E-Cadherin	Goat	1:200	Santa Cruz Biotechnology
F3165	FLAG	Mouse	1:500	Sigma-Aldrich
610921	N-Cadherin	Mouse	1:1,000	BD Biosciences
C19G7	Slug	Rabbit	1:1000	Cell Signaling
sc-7558	Vimentin	Goat	1:200	Santa Cruz Biotechnology
A5316	b-Actin	Mouse	1:1,000	Sigma-Aldrich

Supplementary Table S2. List of antibodies and corresponding working dilutions.
